# Diving-Flight Aerodynamics of a Peregrine Falcon (*Falco peregrinus)*

**DOI:** 10.1371/journal.pone.0086506

**Published:** 2014-02-05

**Authors:** Benjamin Ponitz, Anke Schmitz, Dominik Fischer, Horst Bleckmann, Christoph Brücker

**Affiliations:** 1 Institute of Mechanics and Fluid Dynamics, TU Bergakademie Freiberg, Freiberg, Germany; 2 Institute of Zoology, Rheinische Friedrich-Wilhelms Universität Bonn, Bonn, Germany; 3 Clinic for Birds, Reptiles, Amphibians, and Fish, Justus-Liebig-Universität Giessen, Giessen, Germany; University of Zurich, Switzerland

## Abstract

This study investigates the aerodynamics of the falcon *Falco peregrinus* while diving. During a dive peregrines can reach velocities of more than 320 km h^−1^. Unfortunately, in freely roaming falcons, these high velocities prohibit a precise determination of flight parameters such as velocity and acceleration as well as body shape and wing contour. Therefore, individual *F. peregrinus* were trained to dive in front of a vertical dam with a height of 60 m. The presence of a well-defined background allowed us to reconstruct the flight path and the body shape of the falcon during certain flight phases. Flight trajectories were obtained with a stereo high-speed camera system. In addition, body images of the falcon were taken from two perspectives with a high-resolution digital camera. The dam allowed us to match the high-resolution images obtained from the digital camera with the corresponding images taken with the high-speed cameras. Using these data we built a life-size model of *F. peregrinus* and used it to measure the drag and lift forces in a wind-tunnel. We compared these forces acting on the model with the data obtained from the 3-D flight path trajectory of the diving *F. peregrinus*. Visualizations of the flow in the wind-tunnel uncovered details of the flow structure around the falcon’s body, which suggests local regions with separation of flow. High-resolution pictures of the diving peregrine indicate that feathers pop-up in the equivalent regions, where flow separation in the model falcon occurred.

## Introduction

The peregrine falcon (*Falco peregrinus*) is one of the world’s fastest birds. During horizontal flight, it reaches velocities of up to 150 km h^−1^ ([1], [2]) and even more than 320 km h^−1^ when nose-diving to attack its bird prey (e.g. [3], [4], [5], [6], [7], [8], [9], [10]). Nearly all bird species can alter the shape of their wings and thus can change their aerodynamic properties [11], [12], a concept known as ‘morphing wing’ [13]. During a dive, peregrines also alter the shape of their wings; while accelerating, they move them closer and closer to their body [10]. Several stages can be discriminated: up to about 190 km h^−1^ the falcon shows the classical diamond shape of the wings. This is followed by a tight vertical tuck of the wings up to speeds of 240 km h^−1^. In this flight phase the front part of the wings have a cupped-like profile [14], [15], [16], [9]. At top velocities (up to at least 320 km h^−1^) peregrines build a wrap dive vacuum pack, i.e. the wings are completely folded against the elongated body [17]. Peregrines are not only extremely fast flyers but also maintain remarkable manuverability at high speeds. For instance, during courtship behavior they often change their flight path at the end of a dive, i.e. they turn from a vertical dive into a steep climb. This suggests that peregrines are exposed to high mechanical loads.

Although the nose-diving flight of peregrines has been investigated numerous times, exact measurements of acceleration forces, drag forces, flight path angles and the corresponding aerodynamics of the flow around the body have not been determined. Therefore, we investigated the flight path of peregrine falcons with the aid of high-speed video cameras. The body shape of a diving falcon at maximum flight velocity recorded in a particular experiment (80 km h^−1^) was resembled in a model and the air flow investigated in detail in a wind-tunnel. Here, lift and drag forces were determined for different angles of attack on the falcon model. From these data we deduced the actual flight conditions (angle of attack) regarding to the diving flight. Furthermore, an oil-painting method [18] was used for qualitative flow visualization of near-surface streamlines on the model. The verification of the qualitative visualisation results was done by comparing oil-painting structures with measured velocity fields gained with the itative method named particle image velocimetry (PIV). Comparisons of the results from the model experiments with a real-life falcon provided detailed insights about the aerodynamics and structural adaptations of high speed diving.

## Materials and Methods

### Investigations with Living Falcons

#### Ethics statement

Adult male and female *Falco peregrinus* were used for the flight experiments. Falcons were kept in a voliere at the *Greifvogelstation & Wildfreigehege Hellenthal GbR*. Care was taken according to established falconry practice and current state of the art [19]. Animal husbandry was annually controlled and permitted (*Permission-No.: 60.3/332-63/8, date 15.06.2010, according to 71 § 42 BNatschG*) by the veterinary department (*Kreis Euskirchen, Abteilung 39 - Veterinärwesen & Lebensmittelüberwachung, 53879 Euskirchen, Germany*) and the CITES division of the local authorities (*Kreis Euskirchen, Untere Landschaftsbehörde, 53877 Euskirchen Germany*). All persons responsible for bird training were registered by local authorities (*Kreis Euskirchen, Untere Jagd- und Fischereibehörde, 53877 Euskirchen Germany*). A permission for flight training and handling of the birds (*Permission-No.: 39/591-33, date 22.11.2010, according to § 11 TSchG*) according to national animal welfare law was obtained by the *Greifvogelstation & Wildfreigehege Hellenthal GbR*. All measurements for the present study were performed during routine flights. Therefore, all aspects of the flight trainings remained unchanged from daily routine and were performed according to permission. Our measurements required no additional physical performance, no behavioral restriction and no harmful procedures to the falcon at any time. The measurements were performed at locations belonging to the daily training area of the bird with an additional permission given by the owner of the dam wall Olef-Talsperre (*Wasserverband Eifel-Rur, Eisenbahnstr. 5, 52353 Düren, Germany*) and by the municipal authority (*Gemeindeverwaltung Hellenthal, Rathausstraße 2, 53940 Hellenthal, Germany*).

#### Nose-diving along a dam wall

**Figure 1:**
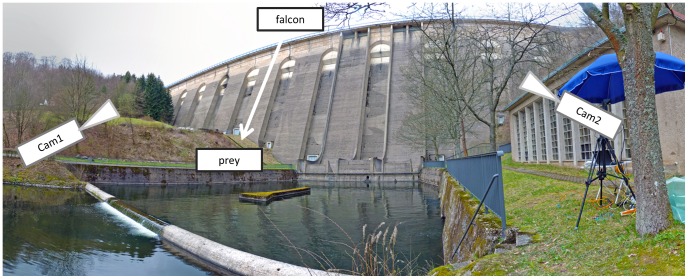
Experimental set-up in front of the dam wall (Olef-Talsperre, Hellenthal, Germany). The two cameras of the stereo system were positioned opposite, separated by a river leaving the dam wall. The features, displayed on the wall enabled us to calibrate the stereo camera system and to exactly determine the position of the diving falcon in the images of both cameras.

Flight experiments were performed in front of the dam wall of the Olef-Talsperre (Hellenthal, Germany). The hight of the dam wall is 60 m, the angle of its inclined surface is 21° relative to the vertical. The front side of the wall points to the south ensuring optimal light conditions for image recordings with high-speed cameras. At noon, shadowing effects were minimal. Furthermore, the dam wall has a well-structured surface featuring horizontal and vertical defined reference markers as well as the painting of a deer in the relevant section for video recording (see arrowhead in Fig. 1). This painting and other wall features enabled us to precisely calibrate the stereo camera system and to determine the exact position of the diving falcon in all video frames. For the nose-dives in front of the dam wall several individuals were used. In total, 35 flights were recorded, one of which was analyzed in detail to obtain acceleration values and body shape information. Due to the professional dive training by the falconer all 35 flights exhibit nearly identical characteristics. Therefore, only one representative flight path is shown in detail. The mass of the investigated falcon was 0.5 kg.

#### Set-up of the camera recording system

**Figure 2:**
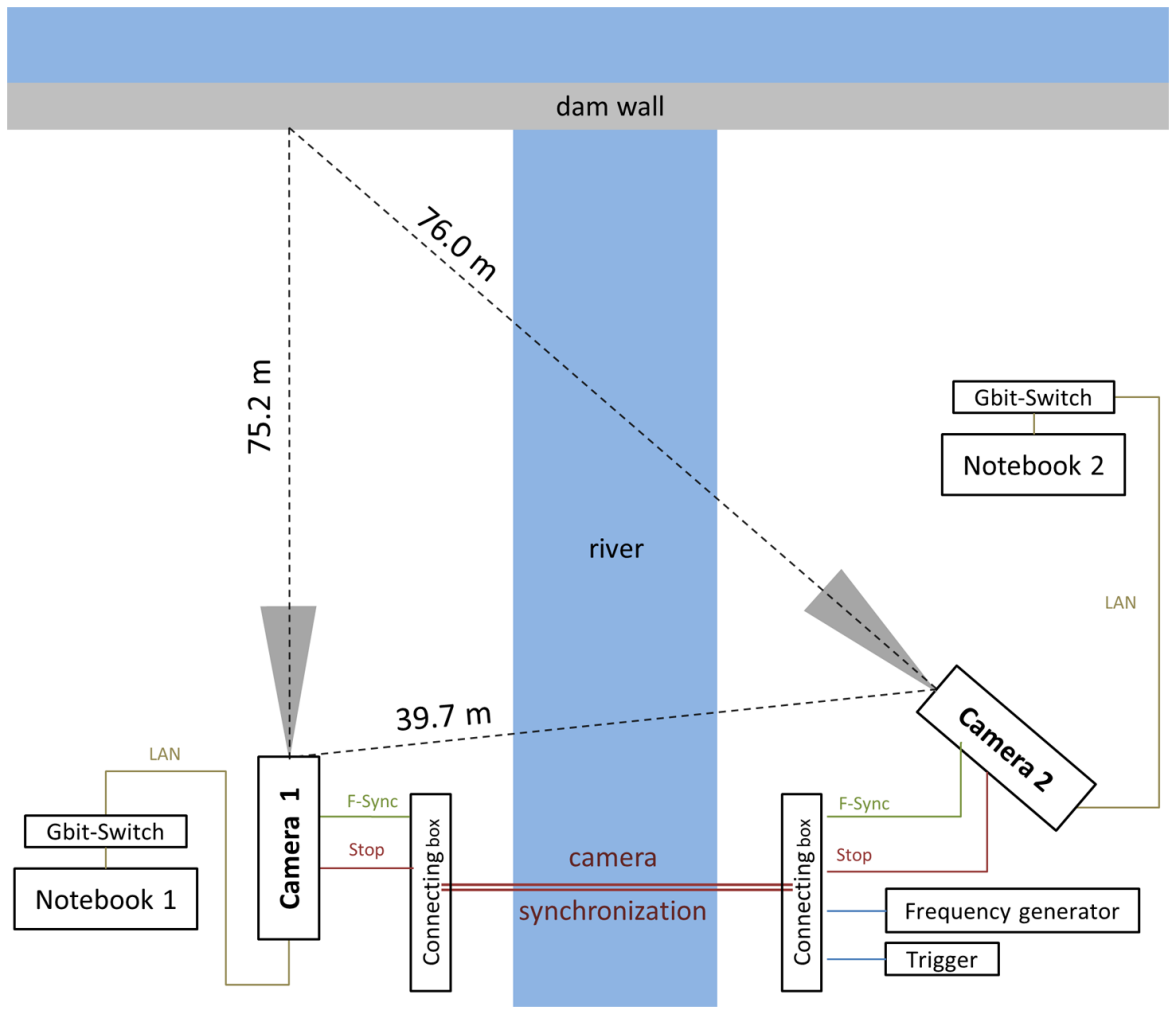
Top-view of the experimental set-up with all measurement components.

A high-speed stereo camera system was used to determine the 3-D flight path trajectories. Fig. 2 shows the spatial arrangement of the equipment in top-view. The stereo system consisted of two monochrome high-speed cameras (Phantom *V12.1* from Vision Research, 1280×800 pixel resolution, pixel size 20 microns, internal memory of 8 Gigabyte RAM) equipped with 35 mm lenses (*AF-S DX NIKKOR)*. The cameras were positioned in the terrain with robust tripods (Gitzo GT5561SGT). The distance between the camera sensors and a reference point on the wall were determined by a laser-based distance sensor (*BOSCH GLM 250 VF Professional)*. For distances up to 250 meters the measuring error was ±1.0 mm. The angular displacement between both cameras was 32.45°; camera #1 was positioned perpendicular to the dam wall (in the horizontal plane). A cable connection between both cameras via BNC (bayonet neill concelman) and TTL (transistor–transistor logic) signal synchronization ensured a simultaneous frame capture. Furthermore, each camera was connected to a separate computer via LAN (local area network) controlling the camera settings. During the measuring procedure the two synchronised cameras captured in ring buffer mode. Cameras where triggered manually when the falcon started its flight. Both cameras stored the recorded images into the internal memory for a few seconds before and after the trigger signal. This method ensured that the entire flight was captured within a recording sequence. Additional information of the falcons body shape and wing contours during the flight were obtained with a third camera (Canon EOS-1D Mark IV, with a 400 mm zoom lens). The position of this camera within the 3-D reconstruction space was also determined and time stamps ensured synchronized observations between the third camera and the both high-speed cameras.

#### Stereo camera calibration and triangulation

The three-dimensional reconstruction of the flight path was achieved by using a stereo-camera system and triangulation. This required a precise calibration of the imaging scene. Within the calibration process the internal parameters of each camera as well as the relative orientation among the cameras were determined [20].

**Figure 3:**
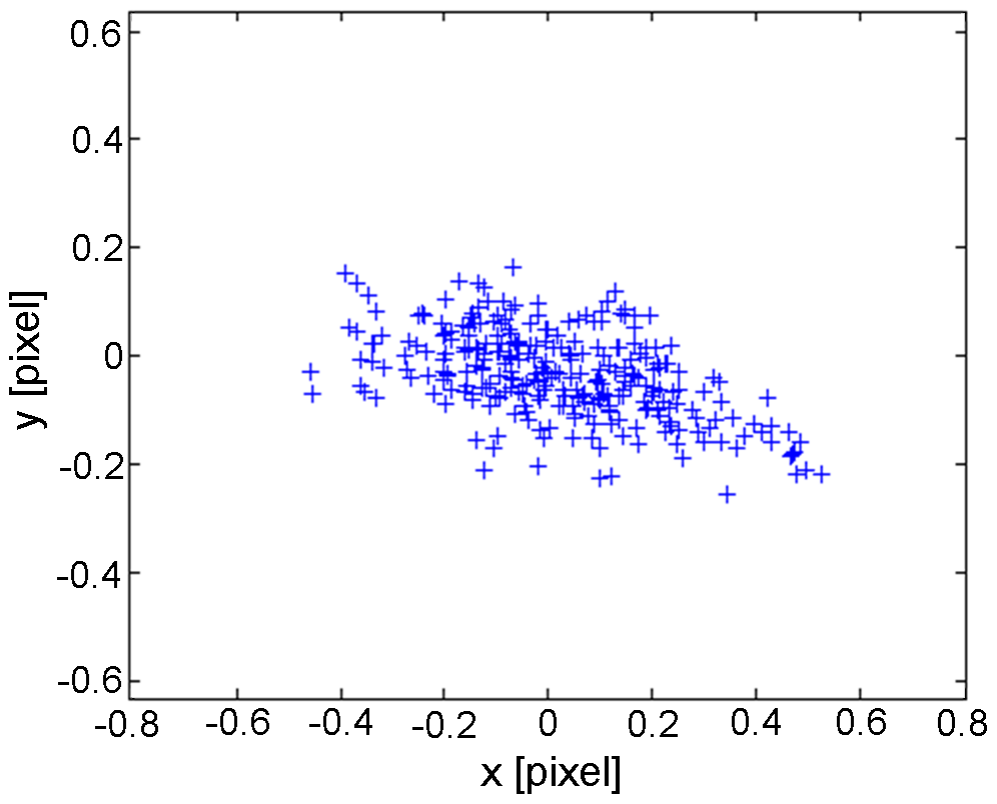
Reprojection error after calibration. The mean values are 0.23 pixel in x- and 0.07 pixel in y-direction.



To quantify the accuracy of the reconstruction method, the reconstructed 3-D points were projected back onto the image plane. The comparison between the back-projected and the original reference point led to the specification of the reconstruction errors. Fig. 3 shows the reconstruction error distribution for all reference points. Mean values of reprojection errors were 0.23 pixel in x- and 0.07 pixel in y-direction. However, the centroid of the falcon can be determined at least with an accuracy of 1 pixel in the image due to the integer values of the pixel size in the camera sensor. A further reconstruction uncertainty was caused by the spatial arrangement of the two cameras. For that reason an error analysis of stereo techniques was carried out according to a procedure given by Lawson [21]. This geometric error model allows the quantification of the displacement error in stereo-systems with angular displacements of the cameras. A relation between the errors of the coordinate components is quantified by the error ratio:
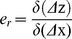
(0.1)where δ(Δx) is the object plane error and δ(Δz) the depth error. The error ratio for the angular set-up of two identical cameras in the centre of the measured field could be approximated by:
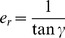
(0.2)where γ is the half-angle between both cameras. Hence, the error ratio of the camera arrangement used in our experiments was:

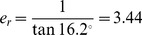
(0.3)Consequently the reconstruction uncertainty in the depth of focus direction δ(Δz) is determined by the transformed equation (0.1):
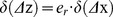
(0.4)


The object plane error δ(Δx) on the camera chip is at least 1 pixel. With a current magnification M = 1/2380 of the camera the image plane error is
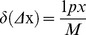
(0.5)which leads to a reconstruction uncertainty in the Z-direction of δ(Δz) = 0.164 m. Thus, the absolute position error is less than half the typical body size of the bird. In comparison, the relative error between successive positions of the bird is less than 1/10 of the body length.

#### Dive training procedures

We tried to keep the flight path of the falcons as reproducible as possible. Therefore, a falconer at the top of the dam controlled the release point, i.e. the point where the falcon started its diving flight. A second falconer at the base of the dam gave the signal to start the falcon and had a lure that attracted its attention.

### Wind Tunnel Investigations

#### Building a one-to-one model

**Figure 4:**
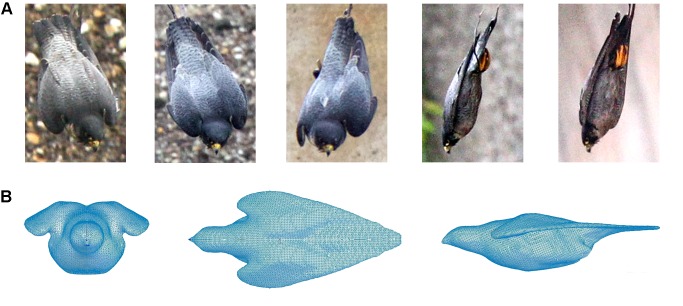
Transformation from a real falcon (A) to a life-size model (B). Pictures from (A) were taken during the diving flights of a peregrine in front on the dam wall. The open wing-shape configuration of the flight was transformed (B).

From the reconstructions of the flight-path and the photographs of the falcon’s body shape we gathered typical images of the falcon while reaching its maximum velocity. The geometry of the falcon in this flight situation was V-shaped (Fig. 4). We compared our photos (Fig. 4A) to images taken from nose-dive investigations reported by the National Geographic Channel [22]. Both, the images provided by the National Geographic Channel and our images displayed the same body shape of a falcon during certain phases of a diving flight. To get the corresponding 3-D body contour of a peregrine during maximum speed in one of our experiments we used the stuffed body of a female peregrine falcon and manually modified its wings until the projections of the body shape closely matched the image of the falcon shown in Fig. 4A. The modified body was fixated and subsequently scanned to attain its 3-D surface contours. Finally, a one-to-one scaled polyvinyl chloride model was fabricated by CNC (computerized numerical control) technology using the acquired 3-D-data (Fig. 4B). Details of the aerodynamic relevant surface areas are given in Table 1.

**Table 1:** Reference areas of the falcon model.

aspect view	reference area
frontal projection area	A_ref,front_ = 0.0123 m^2^
top-view projection area	A_ref,top_ = 0.0411 m^2^

#### Wind tunnel set-up

**Figure 5:**
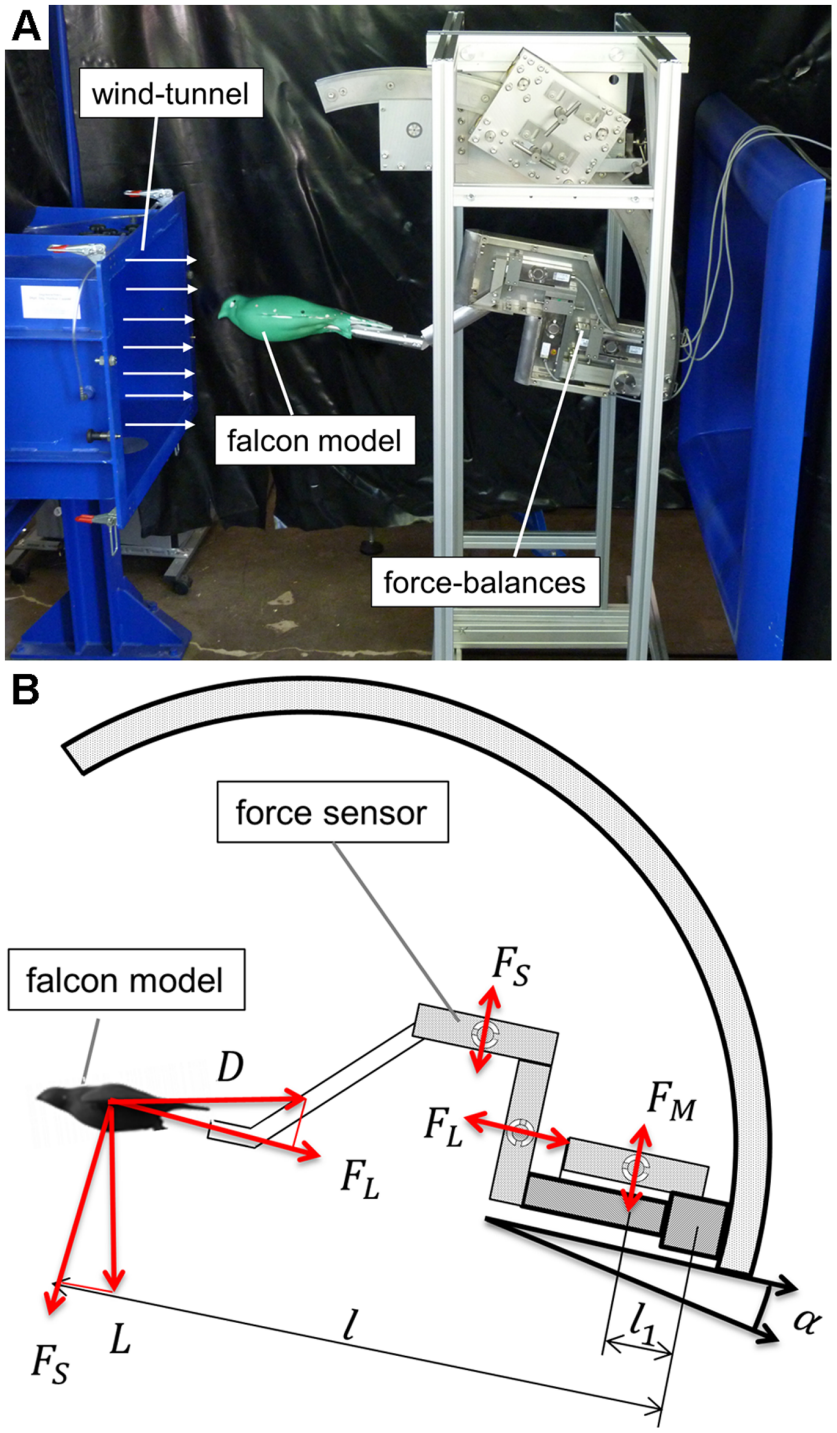
One-to-one falcon model and the measuring device inside the wind-tunnel (A). Functionality scheme of the force-balances (B).

A Göttingen-type wind tunnel was used for the measurements on the falcon model. Specific wind tunnel details are given in Table 2. The model was mounted on the sting of a force-balance device and was placed in the center of the main stream of the wind. The cross-section ratio of the falcon model and the wind tunnel amounts to 4.1%, so that blockage effects are negligible. Fig. 5 shows the experimental set-up (A) and the functionality scheme of the three-component force-balances (B). This measurement device consists of three analog load cells (HBM PW15AH) with an accuracy class C3 (0.02%). The load cells delivered the internal longitudinal force (F_L_) and transversal force (F_S_), from which the lift (L) and drag (D) forces were obtained according to the following equations:

**Table 2:** Wind tunnel specifications.

Test section length	1.30 m
Cross-section	0.30 m^2^ (0.60 m⋅0.50 m)
Level of turbulence intensity	0.04%



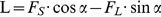
(0.6)

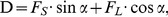
(0.7)where α is the current angle of attack. Table 3 presents the measurement uncertainty of the force balances for different load ranges.

**Table 3:** Force balances error.

load range [N]	error [N]
98.1	0.02
196.2	0.04
490.5	0.10
981.0	0.20

#### Force balances calibration

**Figure 6:**
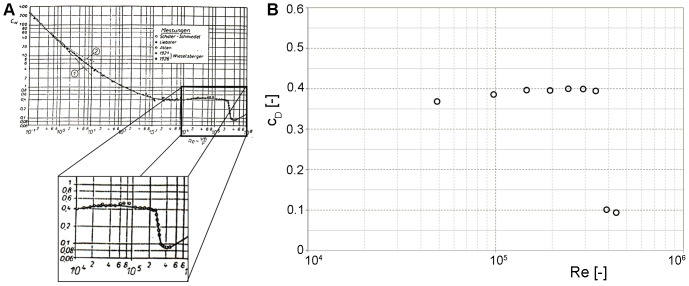
Drag coefficient for flow across a sphere as a function of the Reynolds number. **A:** data from Krause [25]. **B:** measured data by the force balances with typical values documented in the literature as well as the abrupt decrease of the drag at a Reynolds number of about 3.5*10^5^.

Calibration of the force balances was performed using defined loads and using measurements of the drag forces on a sphere in a wind tunnel for different flow velocities. The latter method is well-documented referable to drag coefficient as a function of the Reynolds number (for an example see Achenbach [23] and Schlichting [24]). The Reynolds number quantifies the importance of inertial forces relative to viscous forces and is based on a characteristic length scale. For example, the characteristic dimension of a sphere is defined by the diameter whereas the chord length is used as the characteristic dimension for an airfoil. Our measurements showed an abrupt decrease in drag at a Reynolds number (Fig. 6) where the boundary layer became turbulent. This phenomenon of the so-called ‘drag crisis’ [23], [24] was used to verify the force balances and to demonstrate the independence of measured drag and lift forces.

#### Oil-painting-based flow visualization

An oil-based painting method was used for flow visualization. This method is typically applied to wind-tunnel experiments to visualize the flow structure and the location of possible separation regions at the surface of aerodynamic bodies [18]. The oil-paint consisted of oleic acid, liquid paraffin and titanium oxide. A mixing ratio of 5∶11/1∶6/20 in mass percent was set to gain an optimal viscosity for the present wall shear stress. The entire surface of the model was continuously moistened with a thin mixture film prior to the experiment. After 180 seconds of air flow the near-surface streamlines on the surface of the model became visible.

#### PIV-based flow visualization

For the verification of the oil-painting-based flow visualizations we used the method of particle image velocimetry (PIV) [26]. Hence, velocity fields were measured in planar and parallel layers along the span of the body of the falcon model. The results were obtained by a cross-correlation algorithm and subsequently validated by range and moving average filters of the velocity vectors. Software packages were Dantec DynamicStudio for evaluation and Tecplot360 for visualization of the velocity fields.

## Results

### Flight Experiments

The dive of a wild peregrine is a brief, rare event that takes place at unpredictable places and times, usually at a long distance from the observer. To learn more about the flight characteristics of a diving peregrine we, therefore, used birds that were trained to dive in front of the dam wall of the Olef-Talsperre.

#### Trajectory and wing morphing

**Figure 7:**
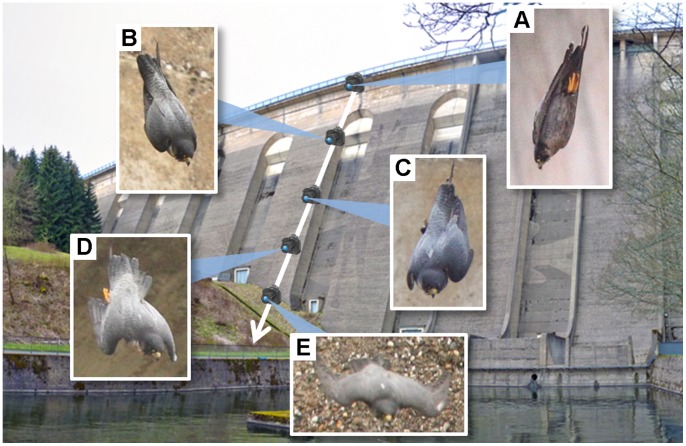
Detail studies for the specific wing shapes.

**Figure 8:**
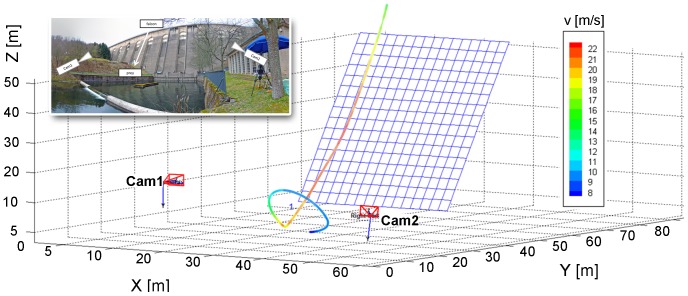
Three-dimensional orientation of the camera set-up based on the spatial calibration. The reconstructed 3-D flight path of the falcon is color-coded with the flight velocity magnitude (red-colored: higher velocities). Maximum velocity during the dive was 22.5 m s^−1^.

**Figure 9:**
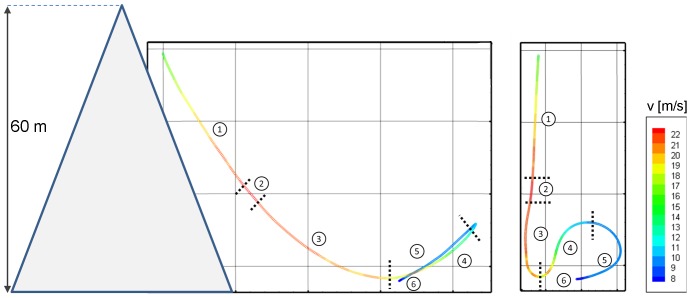
Side-view (left) and front-view (right) of the dam wall and the color-coded trajectory. The six sections of the flight path are acceleration/diving phase (1), transient phase with roughly constant speed (2), deceleration and flight path corrections phase (3), pull out phase (4), landing phase with constant speed (5) and deceleration with touchdown (6).

**Figure 10:**
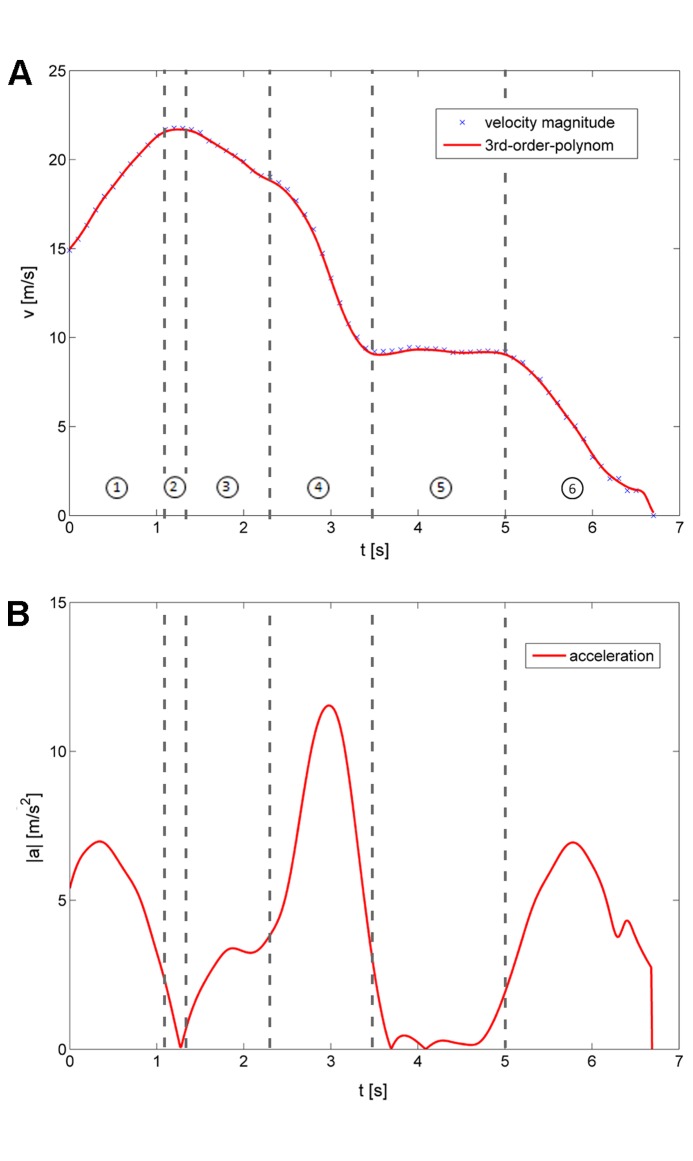
Velocity magnitude (A) and acceleration (B) of the falcon during the time pathway flight. Spline interpolation of the data with the aid of a moving 3^rd^-order-polynomial approximation.

A typical sequence of images taken during a dive at the selected points is given in Fig. 7. Fig. 8 exemplifies the 3-D flight path (sampling rate 400 Hz, duration 6 s) from the beginning of tracking until landing. Flight velocity is color-coded (see calibration bar). In addition, Fig. 8 shows the relative 3-D orientation of the high-speed cameras and the wall. The oblique plane of the wall surface is illustrated with a blue grid (size 2×2 m). The positions of the two high-speed cameras are indicated with the camera symbols. Fig. 9 shows the peregrines flight path in side- and front-view. The characteristics of the entire flight (velocity and acceleration) are depicted in Fig. 10.

Deviations caused by positioning inaccuracies led to an error propagation in the time-derivatives used to calculate the velocity and accelertion values within the trajectory. Therefore, a moving-spline interpolation using a simple central difference scheme was applied to smooth the original data prior to taking the time-derivatives (see Lüthi et al. [27]). This method was used to interpolate the 3-D flight path trajectory, from which the velocity and acceleration values of the falcon were obtained.

The flight was divided in six phases:

1. acceleration/diving phase (0 to 1.1 s);
2. transient phase with roughly constant speed (1.1 to 1.3 s);
3. deceleration and flight path correction phase (1.3 to 2.3 s);
4. pull out phase (2.3 to 3.5 s);
5. landing phase with constant speed (3.5 to 5.0 s);
6. landing phase with deceleration and touchdown (5.0 to 6.8 s).

During phase 1 the falcon accelerated with 6.8 m s^−2^ (its flight velocity increased from 15.0 m s^−1^ to 22.5 m s^−1^). During this phase the falcon covered a vertical distance of 18.55 m. At t = 1.2 s the maximum diving velocity was reached, the flight path angle was 50.75°. This angle θ describes the angular deviation of the flight velocity vector relative to the horizontal (horizontal flight: θ = 0°, vertical dive: θ = 90°) [9].

**Figure 11:**
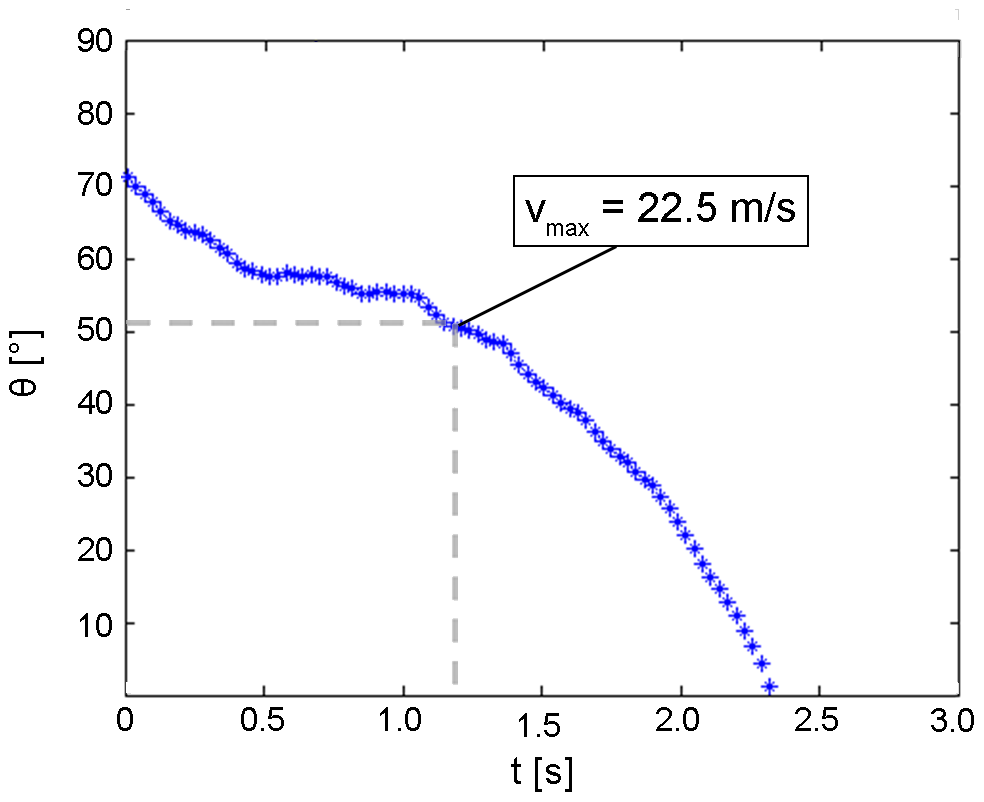
Variation of flight path angle θ in the major tracking phase. The bird starts from almost vertical flight (θ = 90° relative to the horizontal) and still had a flight path angle of θ = 70° when it entered the tracking area (t = 0). The light path angle decreased then continuously until the bird pulled out (θ = 0°) at 2.3 s. The flight path angle is about 50.75° when the bird reached the maximum velocity.

After a short period of nearly constant diving speed (2) the falcon decelerated (3) and within 1.2 s its velocity decreased from 22.5 m s^−1^ to 19.4 m s^−1^. Fig. 11 shows that the flight path angle during the deceleration phase declined from 50.75° to 0°. At the end of the deceleration phase the pull out phase (4) was initiated. This first prominent change of flight direction (pull out) caused a maximum acceleration of 11.5 m s^-^^2^ at t = 2.8 s which is nearly 1.2 times the gravitation constant g (Fig. 10B). A second change of flight direction, which occurred at the pull out phase, led to an acceleration of 9.3 m s^−2^ at t = 3.2 seconds. At the end of the flight, when the falcon completely opened its wings to reduce its velocity and to generate lift, acceleration increased up to 7.0 m s^−2^ for landing. During the pull out phase the falcon significantly decreased its flight path angle θ and most notably its velocity. Furthermore, the falcon changed its body posture from the closed armed wing planform to the open armed wing planform. Hence, the lift forces significantly increased in relation to the drag forces. This is necessary to compensate the intense acceleration forces caused by the decisive changes of the flight path angle.

**Figure 12:**
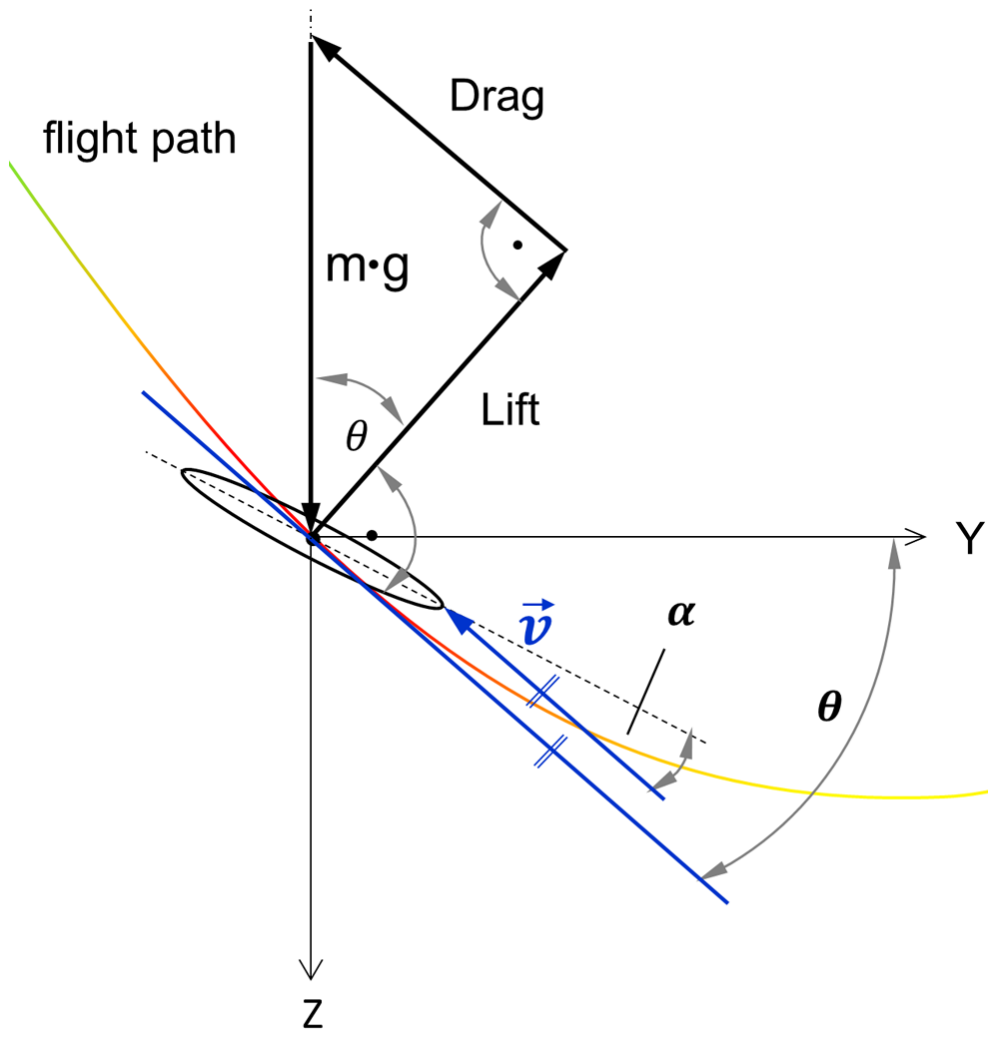
Forces acting at the falcon during diving at maximum speed and zero acceleration. For the given flight path angle θ only one angle of attack α leads to the fulfilled condition of equilibrium.

Fig. 12 depicts the forces acting on the falcon during the diving flight at the maximum speed reached in our experiment. This phase with zero acceleration allowed us to apply the condition of equilibrium of the sum of aerodynamic forces with the gravitational force that should cancel out each other to zero. In addition, this is the only situation where the body shape of the falcon model fits to flight aerodynamics under steady state conditions and constant angle of attack. The latter is, however, not known a priori and must be derived from the polar diagrams of an aerodynamically equivalent model of the falcon in the wind tunnel. The following condition needs to be met to cancel the force in Y-direction:
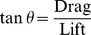
(0.8)


At this point of maximum speed the 3-D trajectory of the flight path angle θ was 50.75°. Accordingly, the ratio between drag and lift forces must be 0.817. With the aid of the aerodynamic polar diagram of the falcon (see below), we could derive the angle of attack α where this specific ratio is reached. Therefore, we carried out additional detailed force measurements of the falcon model in the wind tunnel at varying angle of attack.

### Wind Tunnel Investigations with a Life-size Model

#### Lift and drag coefficients

**Figure 13:**
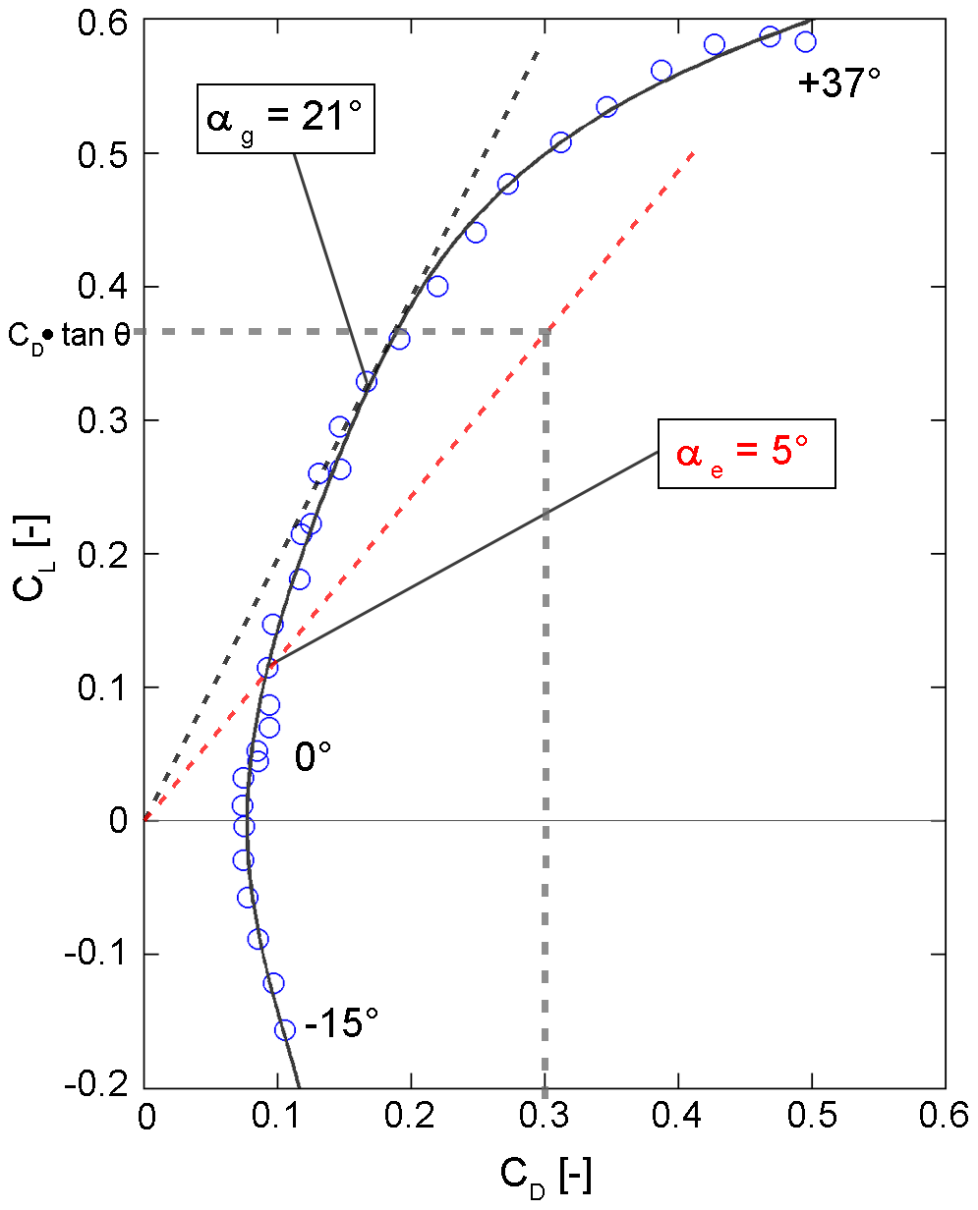
Polar diagram of the falcon model for a velocity of 22.5^−1^. The angles of attack α varied from −15° to +37°. The increase of the pole line (black dashed line) of the open wing shape is m = 1.93 for the best gliding angle of α_g_ = 21°. The increase of the linear function (red dashed line) for the equilibrium condition is m = 1/tan(θ) = 1.22. Hence the intersection of this linear function with the polar curve leads to an angle of attack α_e_ = 5° in the free flight situation.

With the measured lift and drag forces the associated coefficients could be determined as follows
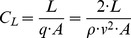
(0.9)and



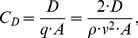
(0.10)where q is the dynamic pressure, ρ is the mass density of the fluid, v is the free stream velocity and A is the reference area of the wing, in this case the top-view projection area (A_ref,top_) of the falcon model. The functional relationship between lift and drag coefficients for varying angles of attack led to polar diagrams for airfoils named after Otto Lilienthal. Fig. 13 shows the polar diagram for the falcon model at a free stream velocity of 22.5 m s^−1^ (the maximum speed reached during the dive). The angle of attack was varied between −15° and +37°. With the previously calculated flight path angle θ = 50.75° the linear relationship
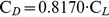
(0.11)is given. Thus, the slope of this function in the polar diagram is defined by
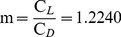
(0.12)


**Figure 14:**
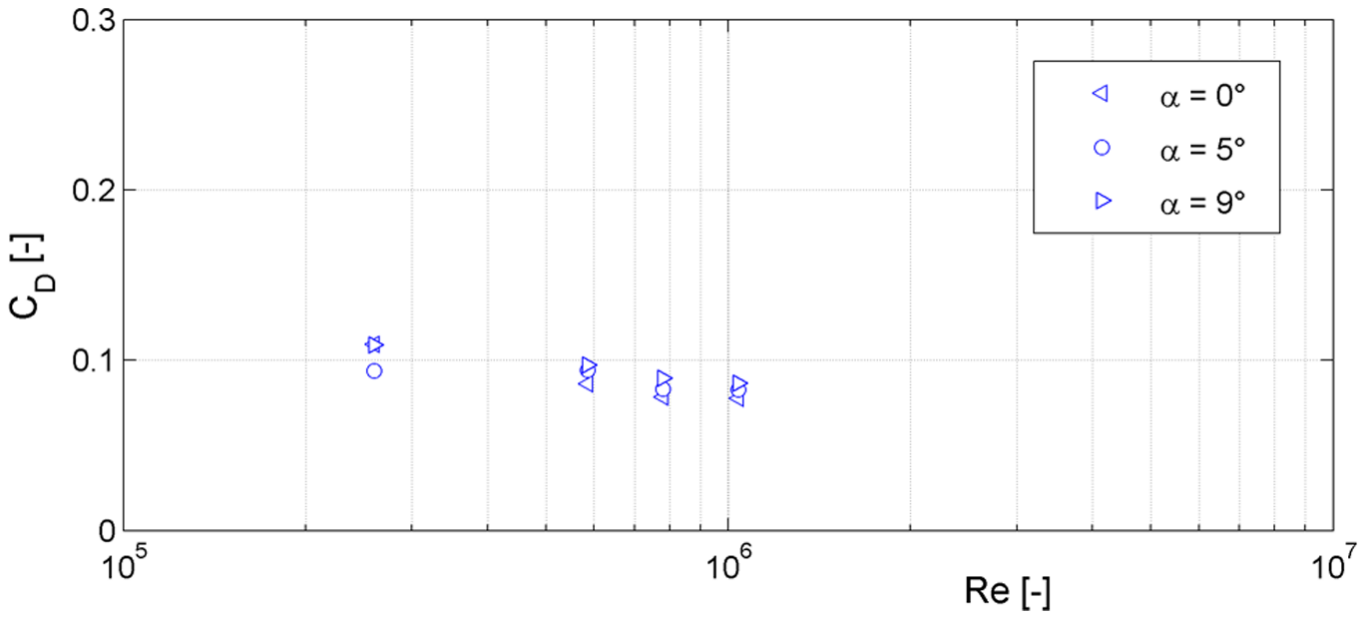
Drag coefficient vs. Reynolds number of the falcon model for different angles of attack α. Measurements were done for different Reynolds numbers [Re = 260 000 (v = 10 m s^−1^), Re = 585 000 (v = 22.5 m s^−1^), Re = 780 000 (v = 30 m s^−1^) and Re = 1 040 000 (v = 40 m s^−1^)].

The intersection of this linear function with the measured polar curve leads to an angle of attack of α = 5°. This was the angle of attack in the diving experiment, i.e. when the falcon reached the maximum velocity with its V-shaped wings. The corresponding values of lift and drag coefficients for the equilibrium flight phase at an angle of attack of α = 5° are given in Table 4. As a reference, the values at an angle of attack of α = 0° are also given. Additional wind tunnel tests with varying flow speeds were carried out to investigate the relevance of turbulence on the two forces. Fig. 14 illustrates the drag coefficients for different Reynolds numbers. The Reynolds number for the maximum diving velocity of v = 22.5 m s^−1^ and the characteristic length of the falcon model L_ch_ = 0.4 m amounted to

**Table 4:** Lift and drag coefficients for a parallel direction of flow (α = 0°) and an angle of attack α = 5° for a velocity of 22.5 m s-1.

	α = 0°	α = 5°
**Lift coefficient C_L_**	0.0445	0.0870
**Drag coefficient C_D_**	0.0860	0.0941



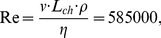
(0.13)where ρ is the mass density and η the dynamic viscosity of the fluid. Hence, the boundary layer flow around the falcon was turbulent at this speed. The measured drag coefficient only weakly depended on the Reynolds number in a range between Re = 260 000 (v = 10 m s^−1^) and Re = 1 040 000 (v = 40 m s^−1^).

#### Near-surface flow visualization

**Figure 15:**
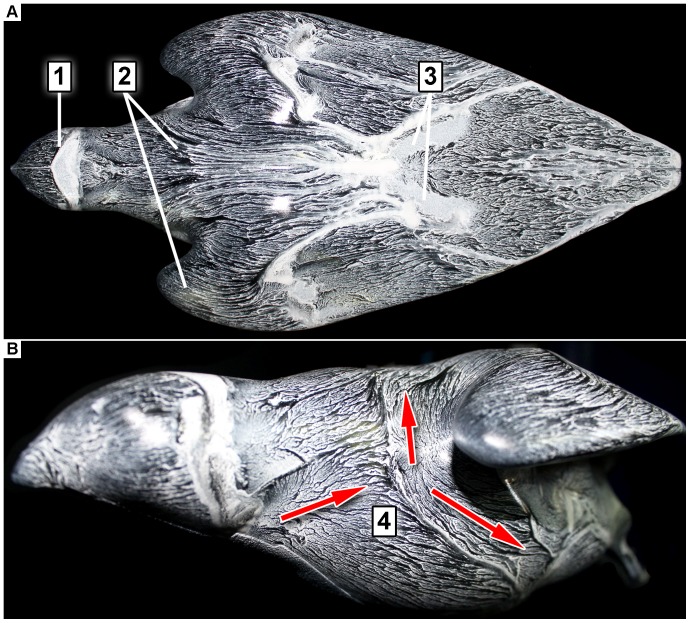
Flow visualization on the surface of the falcon model via oil-based painting. **A:** top-view, **B:** frontal side-view. (Re = 5.8 10^5^, angle of attack α = 5°, flow direction is from left to right).

Fig. 15 shows the oil-painted falcon model in the wind tunnel for the equilibrium flight situation. The angle of attack was 5°. As can be seen in Fig. 15 the ‘surface streamlines’ on the model’s surface uncover areas with different topological patterns of the thin liquid film. These structures can be used to draw conclusions about the flow over the body (e.g. stagnation points, flow separation and flow reattachment) [28], [29]. Area (1) shows the flow structure over the head. The surface streamlines near the frontal part of the head are aligned in flow direction. Downstream to this region there is a brighter region where the oil accumulates. This indicates a local flow separation where oil transportation was strongly reduced. Immediately further downstream the flow re-attaches again. In the region of the falcon’s neck a darker zone, which indicates a higher local velocity, is visible (region 2). Between region 2 and 3 the surface streamlines are arranged quite orderly over the V-shaped wing. A higher intensity of oil around region 3 indicates another small region where local flow separation occurred. Region 4 in the lateral view of the model shows the formation of a separating streamline and a stagnation point in the groove between body and wing shoulder. Consequently, the oil transportation at this point is spread unevenly over different parts of the falcon’s body.

**Figure 16:**
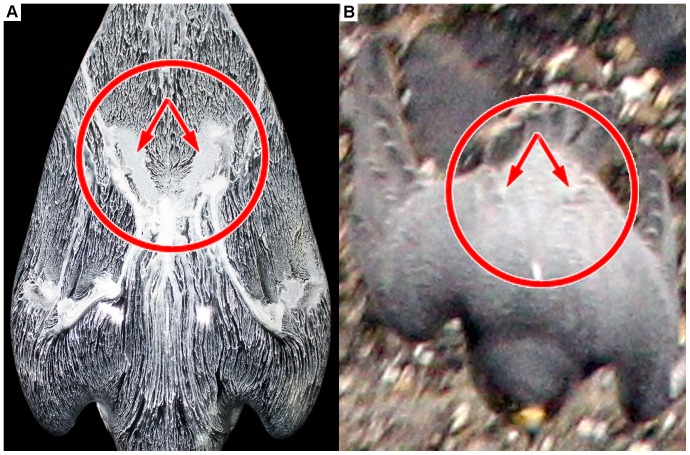
Flow visualization on the surface of the falcon model via oil-based painting (A) and the living creature at the identical flight position (B). The area of the red circle shows a homogenous white colored oil-painting. This indicates a local separation of flow. The falcon on the right-hand side shows small feathers which are popped-up from the falcon body in this region. It is assumed that this specific arrangement of the feathers prevents local flow separation on the falcon body in nature.

**Figure 17:**
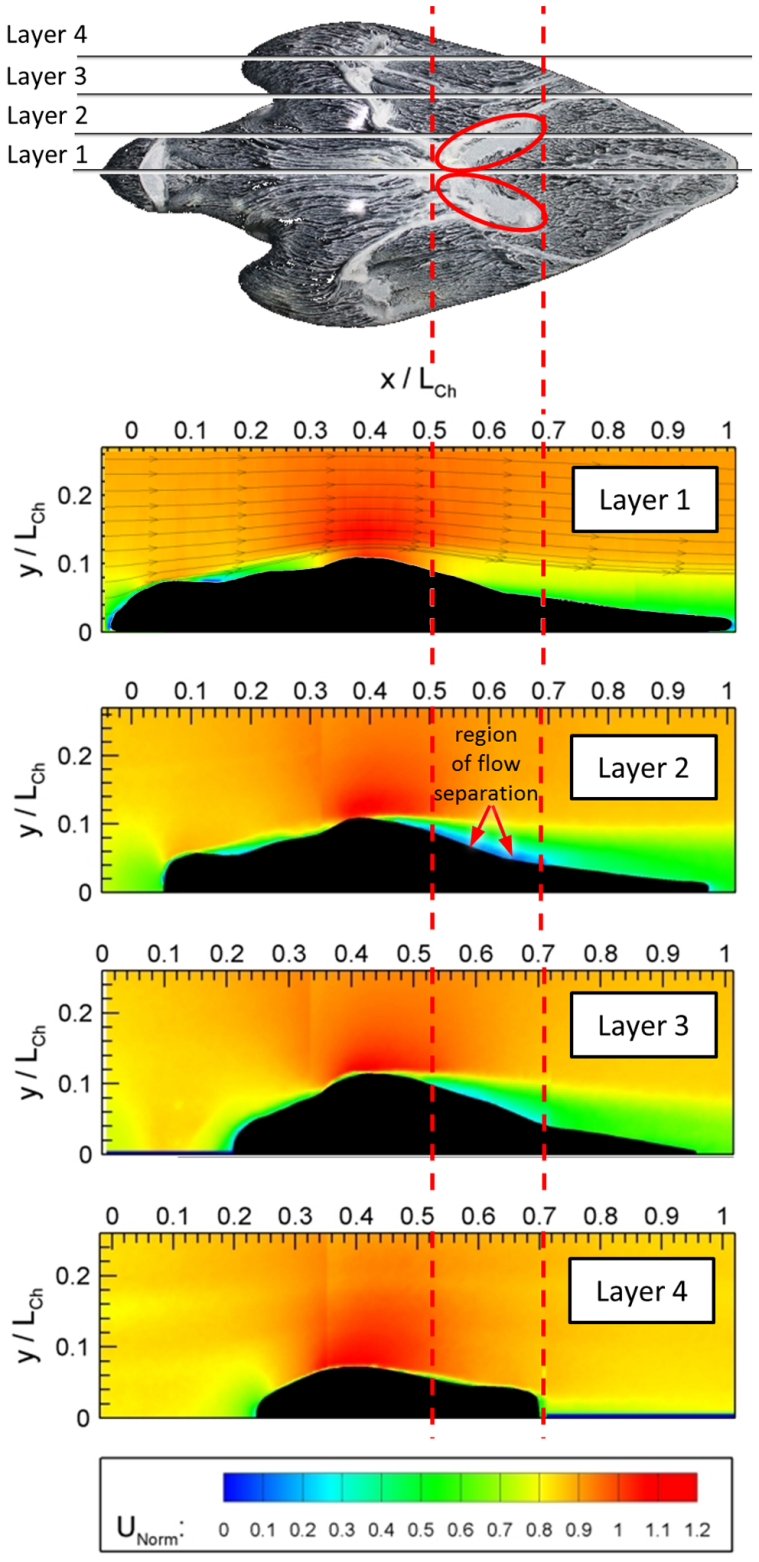
Flow visualization in four cross-sections of the falcon model via particle image velocimetry (PIV). Layer one goes through the plane of symmetry whereas layers two to four have an offset in each case of 14-painted top-view of the model shows that only layer two crosses the region where a higher intensity of oil indicates area where local flow separation is seen. Exactly in this region the PIV results show a dead water region in comparison to the other layers.

Fig. 16 compares the near-surface flow visualization of the model (A) with a picture of the diving falcon for the identical flight situation (B). Within the red circle (Fig. 16A) a homogenous white colored oil distribution is visible. This indicates a local flow separation. Fig. 16B shows that feathers of the diving falcon popped-up in the same region. Note, that these feathers are usually fitted tight to the body. In order to gain more quantitative details in this region we analyzed the flow on the suction side of the model by using planar particle image velocimetry (PIV). Fig. 17 shows the flow by means of contours of constant velocity magnitude in four certain cross-sections (layers one to four) of the falcon model. Layer one goes through the plane of symmetry whereas layers two to four have an offset in each case of 14 mm in relation to the previous layer. The area of interest, where flow separation was seen by near-surface flow visualization is marked with vertical red-colored dashed lines. The oil-painted top-view of the model (Fig. 17, top) shows that only layer two crosses the region where a higher intensity of oil indicates area where local flow separation is seen. Exactly in this region the PIV results show zero valued velocities in comparison to the other layers.

## Discussion

This study presents the results obtained from a diving peregrine falcon and from flow and force measurements performed in a wind tunnel using a falcon model that scaled 1∶1 to the real life animal. Combining both studies enabled us to determine the actual angle of attack during a nose-dive at maximum speed. For the study, the 3-D flight path trajectory of a diving peregrine was recorded; a high-resolution camera simultaneously captured images of the body and wing shapes. During a dive in front of a 60 m high dam the falcon reached a maximum velocity of 22.5 m s^−1^. For equilibrium flight conditions (maximum speed, zero acceleration) the flight path angle was θ = 50.75°. Using high-resolution images obtained from the diving falcon we built a real-size model of a peregrine falcon. The typical contour of a falcon’s body is V-shaped with openings at the shoulders and a tip at the tail. Combining the outdoor measurements with the wind-tunnel tests allowed us to determine the actual angle of attack at α = 5° for this flight condition. For a flight velocity of 22.5 m s^−1^ and an angle of attack of 5° the lift coefficient was C_L = _0.0870 and the drag coefficient was C_D = _0.0941 (Table 4). This was equal to a 96% increase for lift and a 10% increase for drag compared to the values obtained at a zero angle of attack (α = 0°).

The equilibrium flight conditions were also investigated with regard to the details of the flow topology along the model. Oil-painting based flow visualization and PIV results revealed discrete regions of flow separation on the head and in the last third of the body. By relating these findings to the corresponding flight situation of a diving falcon it became apparent that several small feathers popped-up at the same positions where the flow visualization methods indicated a flow separation on the model. Therefore, the pop-up feathers most likely act as passive flow control units that prevent flow separation (see [30], [31]).

Due to the low height of the dam wall the falcons only reached velocities of up to 80 km h^−1^ in our experiments. As expected, they showed the classical diamond shape but not the wrap dive vacuum pack [17], i.e. their wings were not completely laid against their body. Further experiments are needed to discover more about the aerodynamics of falcons that dive with speeds in access of 80 km h^−1^.

## Conclusions

Flight-path measurements on living falcons and simultaneous high-resolution imaging under well-controlled conditions enabled us to investigate details of the special flight conditions necessary for maximum diving speed in our experiment. The focus of the detailed flow studies using a falcon model was on the equilibrium condition where acceleration was zero and forces on the falcon summed up to zero. Parameters like maximum diving velocity, flight path angle and shape of both body and wing for this particular situation of the flight path were prerequisites to investigate a life-sized model of a peregrine in a wind tunnel at the correct flow conditions and angle of attack. The major outcomes of this experiment are the following:

- The typical shape of both the body and the wing at maximum diving speed (in this experiment) shows a V-type structure with the open end between the shoulders and the tip at the tail of the body. The leading edge of the wing is not straight but has a wavy structure with grooves in the gaps between the neck and both shoulders. In this region separating streamlines and a stagnation point are uncovered with the aid of near-surface flow visualization.
- The real life images show that feathers stick out (pop-up) during the dive at exactly the same regions on the upper surface (suction side) of the wing, where the oil-painting-based flow visualization studies in the wind tunnel experiment revealed local flow separation. In addition, PIV measurements verified identical regions of flow separation. It is assumed that the presence of the popped-up feathers prevents this local flow separation during the diving flight of the peregrine falcon, similar to what has been shown to occur in experiments and simulations on airfoils covered with self-adaptive flaps (see [32], [33], [31], [34], [35], [36]).
